# Editorial: Mechanisms of Action in Neurodegenerative Proteinopathies

**DOI:** 10.3389/fnins.2022.968994

**Published:** 2022-06-30

**Authors:** Sean J. Miller, Selina Wray, Rita Sattler, Can Zhang

**Affiliations:** ^1^Pluripotent Diagnostics Corporation (PDx), Molecular Medicine Research Institute, Sunnyvale, CA, United States; ^2^Department of Neurodegenerative Disease, UCL Queen Square Institute of Neurology, University College London, London, United Kingdom; ^3^Department of Translational Neuroscience, Barrow Neurological Institute, Phoenix, AZ, United States; ^4^Genetics and Aging Research Unit, Department of Neurology, McCance Center for Brain Health, MassGeneral Institute for Neurodegenerative Disease, Massachusetts General Hospital and Harvard Medical School, Charlestown, MA, United States

**Keywords:** proteinopathies, neurodegeneration, neuropathology, Alzheimer's disease, amyotrophic lateral sclerosis, frontotemporal dementia

Proteinopathies, or the neuropathologies primarily caused by the abnormal aggregation of specific proteins in the brain, are a defining factor in most, if not all, neurodegenerative disorders. There are currently no cures or disease-modifying therapies for most neurodegenerative disorders caused by proteinopathies. The mechanisms leading to protein aggregation, and linking proteinopathy to downstream neurodegeneration, are not fully understood. Therefore, a better understanding of these mechanisms and the relationship between protein aggregation and neurodegeneration in different cell types and model systems is imperative for our understanding of neurodegenerative disorders and the development of novel therapeutics.

The Research Topic on “Mechanisms of Action in Neurodegenerative Proteinopathies ” in *Frontiers in Neuroscience* is developed with the primary goal to discuss and review the current state of the field and to provide new evidence with respect to proteinopathies. Focusing on pathological and molecular mechanisms linking protein aggregation to neurodegeneration, a series of 7 articles in this Research Topic are included and previewed below.

The article by Gagné et al. investigates the spatiotemporal expression pattern of an RNA-binding domain containing protein, the heterogeneous ribonucleoprotein A1 (hnRNP A1) and its splicing variant hnRNP A1B in mice. The results show that both isoforms are differentially expressed across tissues with distinct localization profiles, suggesting their specific subcellular functions with potential contribution to related disease heterogeneity in neurodegeneration. Furthermore, this article supports the notion that RNA binding proteins (RBPs) play pivotal roles in cellular growth, homoeostasis and survival and are tightly regulated. Future studies are required to better understand their contribution to physiology and pathology of neurodegenerative disorders.

Next, Garrett and Niccoli summarize mechanisms of impaired glucose metabolism in frontotemporal dementia (FTD), the second most common early onset dementia following Alzheimer's disease (AD). Although well-characterized and hallmarked by antero-temporal degeneration in the brain, the pathogenesis of FTD remains to be further elucidated. In this article, the authors provide a comprehensive review of recent discoveries and current understanding of FTD with a particular association with glucose metabolism underlying disease.

Despite considerable evidence showing the loss of TDP-43 protein homeostasis and aggregation underlying amyotrophic lateral sclerosis (ALS) and FTD, the molecular mechanisms that regulate protein aggregation require further studies. TDP-43 is also an RNA binding protein, and tightly controls its own expression levels through a negative feedback loop, involving TDP-43 recruitment to the 3′ untranslated region (3'-UTR) of its own transcript, which needs further mechanistic studies. To this end, Koehler et al. perform a study that further investigated the mechanisms by which TDP-43 protein may perform autoregulation in association with the pathogenesis of ALS and FTD.

Additionally, Zhang et al. investigate the association of the protein-quality-control (PQC) protein ubiquilin-1 with AD using cell and animal models. Genetic analysis has identified ubiquilin-1-encoding *UBQLN1* gene as an AD candidate gene and ubiquilin-1 levels reduce with AD progression. The effects of ubiquilin-1 on cellular physiology as well as molecular events require further investigation. By studies in this domain, this article supports not only a loss-of-function mechanism of ubiquilin-1 in association with AD, but also supports the significance of targeting ubiquilin-1-mediated PQC as a potential therapeutic strategy for AD.

Despite decades of research, there is still no cure for Parkinson's disease (PD), the most common movement disorder and the second most prevalent neurodegenerative disease after AD. The complicated intricacies underlying PD are still needing further elucidation. Recently, neuroglia has become recognized as key players in the health and disease of the central nervous system (CNS). Due to the broad and keen research interest in this specific domain, Miller et al. contribute a review article on neuroglial senescence and α-synucleinopathy as well as therapeutic potential of senolytics for PD.

One key area for understanding and potentially curing AD is a focus on amyloid precursor protein (APP) and its cleavage fragment Amyloid-β (Aβ), which may drive a subsequent cascade of pathological events of AD (aka “amyloid cascade”) and eventually result in major hallmarks in AD brains, including amyloid plaques and neurofibrillary tangles. Reducing APP expression is an attractive approach for AD treatment and prevention which needs an urgent review due to a large body of knowledge and discoveries in this area. Dedicated in this area, Gabriele et al. contribute a comprehensive review article describing the knockdown of APP with concentrations on biological consequences and clinical opportunities.

Lastly, to further broaden the understanding of AD pathogenesis, Alawode et al. present a review article which extends the standpoint of “amyloid cascade” hypothesis by linking to disease biomarkers. Although it is well-known that deposition of Aβ in the brain parenchyma is a crucial initiating step necessary for progression of AD, it is only recently that methodologies and instruments are developed to trace proteins in blood and cerebrospinal fluid (CSF) with high-sensitivity. Here, the authors discuss the potential effects of Aβ on surrounding tissues, and consider the potential of monitoring Aβ-related responses using compelling biomarkers to phenotype AD and potentially stratify AD patients.

Above all, the articles collected in this Research Topic are able to cover a diverse research scope, including the characterization of underlying mechanisms that link protein aggregation to neurodegeneration and therefore provide a better understanding of common neurodegenerative disorders; the use of disease modeling for neuropathological hallmarks; and finally the evaluation of therapeutic approaches to prevent or ameliorate proteinopathies using biochemical, genetic, and cellular methodologies.

In closing, we would like to thank all of our contributing authors and reviewers for their valuable expertise, suggestions, and effort for making our collection possible. These articles warrant more committed research in the future that should be conducted to better elucidate the underlying mechanisms of proteinopathies in neurodegeneration. It is our hope that the collection of these articles may provide the readers with useful references that may help advance and stimulate future studies in this domain aiming to ultimately understand complex mechanisms underlying proteinopathies and to develop useful therapeutics.

## Author Contributions

All authors listed have made a substantial, direct, and intellectual contribution to the work and approved it for publication.

## Conflict of Interest

SM is the Chief Executive Officer & Founder of Pluripotent Diagnostics Corp. The remaining authors declare that the research was conducted in the absence of any commercial or financial relationships that could be construed as a potential conflict of interest.

## Publisher's Note

All claims expressed in this article are solely those of the authors and do not necessarily represent those of their affiliated organizations, or those of the publisher, the editors and the reviewers. Any product that may be evaluated in this article, or claim that may be made by its manufacturer, is not guaranteed or endorsed by the publisher.

